# Combined Robotic Transoral–Transhyoid Approach for Salvage Surgery for Recurrence of Base of Tongue Squamous Cell Carcinoma After Primary Radiation

**DOI:** 10.1007/s12663-024-02227-z

**Published:** 2024-08-11

**Authors:** Simon E. Thurnheer, Christof Buhl, Gunesh P. Rajan, Grégoire B. Morand

**Affiliations:** 1 Department of Otorhinolaryngology, Head and Neck Surgery, Luzerner Kantonsspital, Lucerne, Switzerland; 2 Faculty of Health Sciences and Medicine, University of Lucerne, Lucerne, Switzerland; 3 Department of Otorhinolaryngology, Head Neck Surgery, University Hospital Zurich, Frauenklinikstrasse, 248091 Zurich, Switzerland; 4 University of Zurich, Zurich, Switzerland

**Keywords:** Squamous Cell Carcinoma of Head and Neck, Robotic Surgical Procedures, Salvage Head and Neck Surgery, Margins of Excision, Morbidity, Follow-Up Studies

Salvage surgery for persistent or recurrent oropharyngeal squamous cell carcinoma (OPSCC) after primary (chemo) radiation is usually associated with significant morbidity and/or difficulty to achieve clear resection margins [1, 2].

**Fig. 1:**
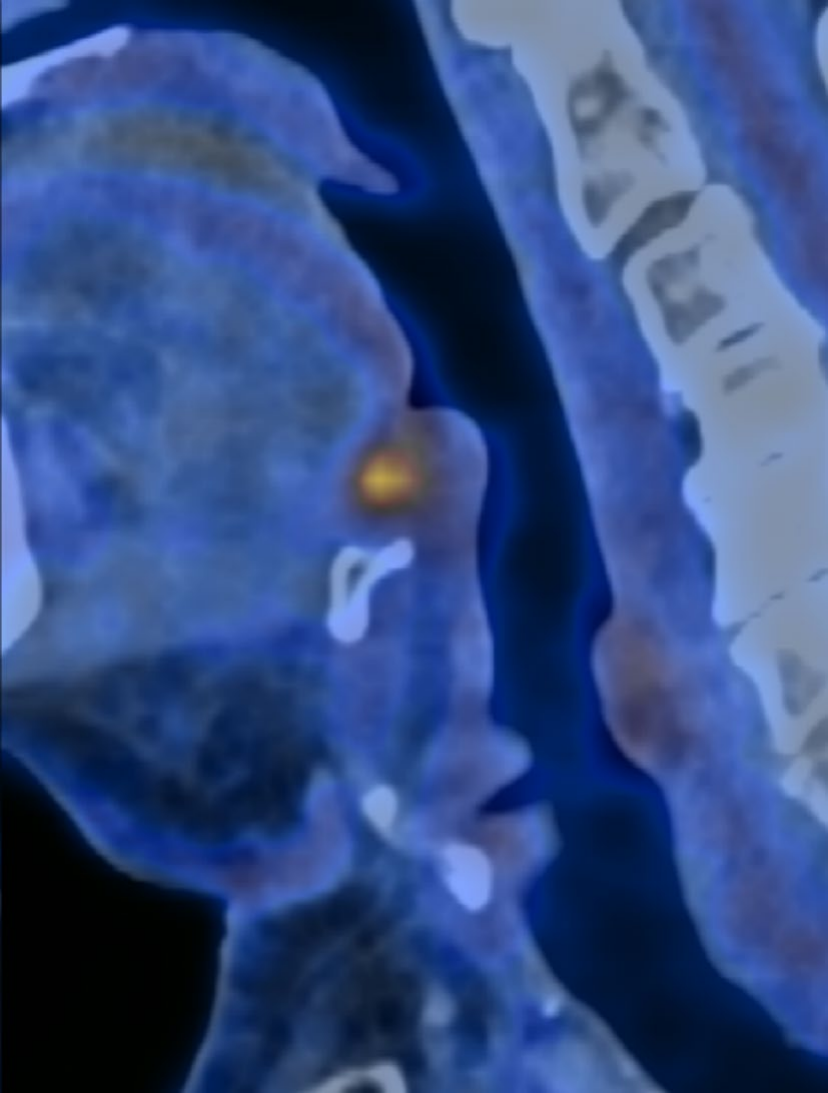
Sagittal PET-CT of recurrent cancer

We present a case of an 80-year-old patient with *p*16-negative base of tongue OPSCC who presented with recurrent disease after primary curative hypofractionated radiotherapy (Fig. 1). Our novel combined transoral–transhyoid robotic approach [3, 4] combined transoral robotic surgery (TORS) with transcervical mobilization of the hyoid in order to optimize exposure of the base of tongue as well as assuring clear deep “bony” margins toward the body of the hyoid which was resected en bloc with the base of tongue.

**Fig. 2:**
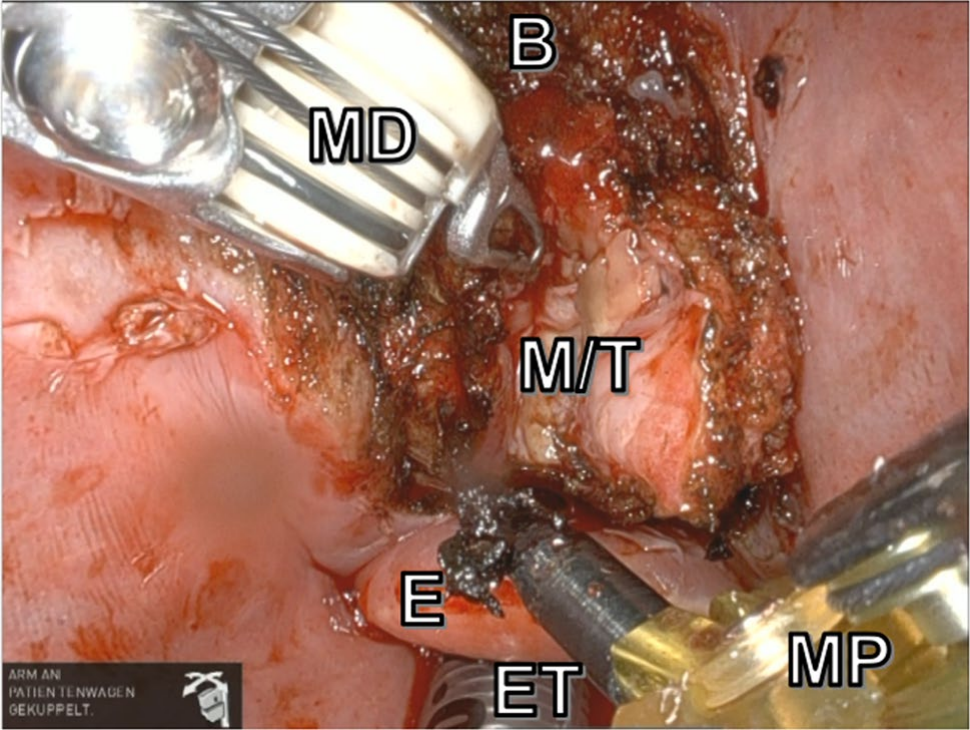
Intraoperative view before transcervical push of the hyoid body. M/T, mucosa over tumor/tumor; ET, endotracheal tube; E, epiglottis; B, base of tongue; MD, Maryland dissector, MP: monopolar

**Fig. 3:**
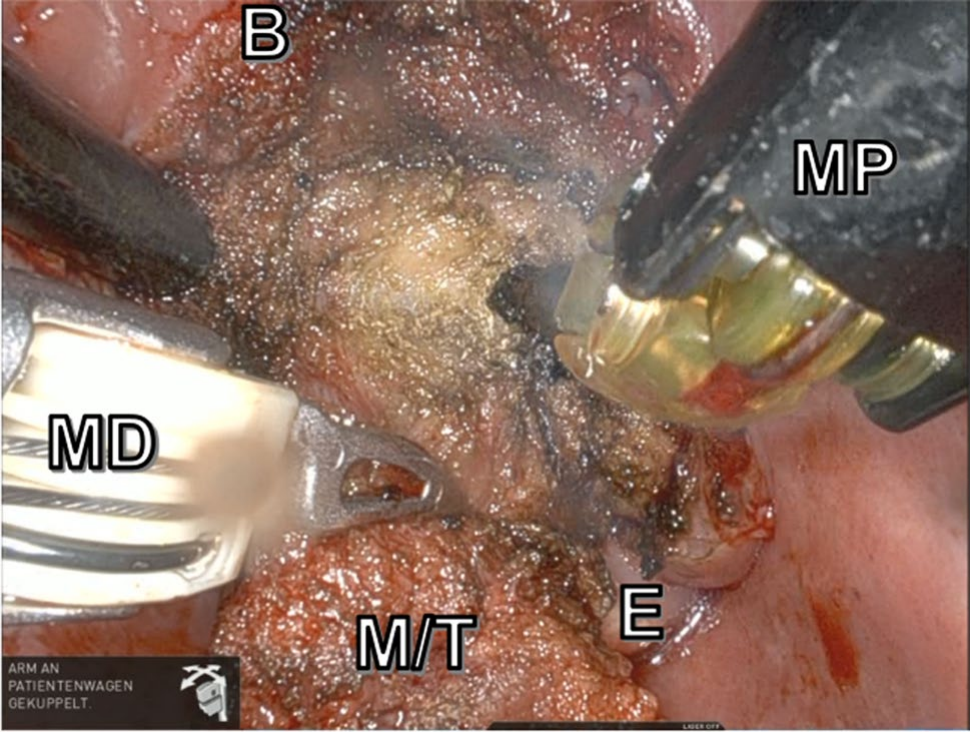
Intraoperative view after transcervical push of the hyoid body. M/T, mucosa over tumor/tumor; E, epiglottis; B, base of tongue; MD, Maryland dissector, MP: monopolar

In addition to a conventional TORS setup for base of tongue resection, we prepped and draped the neck and access the body of hyoid through a 3 cm midline incision. The body of the hyoid bone was exposed, freed from its inferior muscular attachments and cut just lateral of the lesser corns. Care was taken not too free the hyoid body superiorly in order not to compromise the deep margins of the resection. Generous mucosal margins were taken on both sides with care not to injure the lingual arteries and hypoglossal nerves. Meanwhile one assistant was providing suctioning of fumes intraorally, and another assistant was mobilizing the hyoid bone and improving TORS exposure with the help of an Allis clamp (Fig. 2 and Fig. 3).

**Fig. 4:**
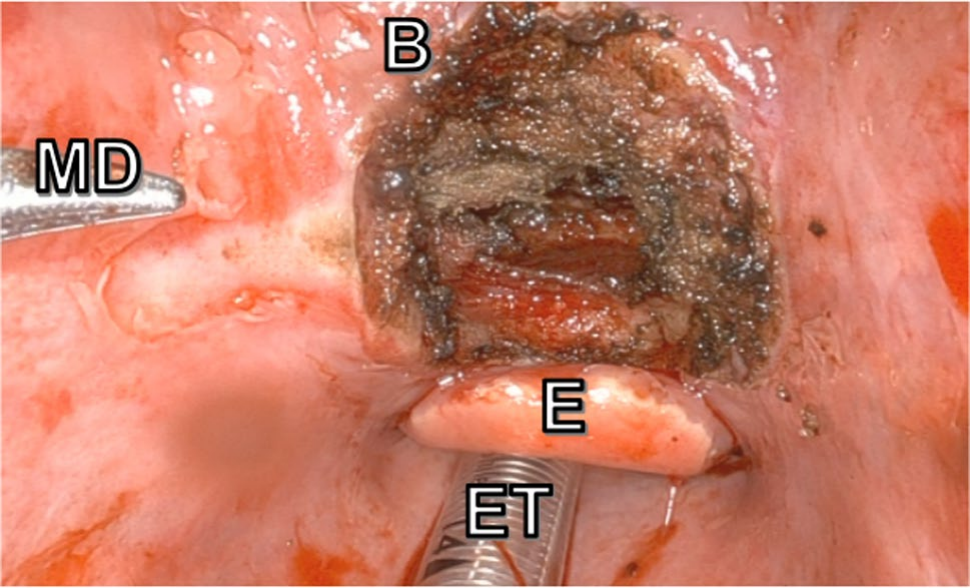
Soft tissue defect after resection. ET, endotracheal tube; E, epiglottis; B, base of tongue; MD, Maryland dissector

The resection resulted in a through and through defect of about 3 cm in diameter centered in the midline (Fig. 4). A five-layer closure was obtained as the intrinsic muscle of the tongue was closed in the coronal plane, and the hyoglossal muscle was closed in a transverse plane, followed by the strap muscles, plastysma and skin closures.

Postoperatively a nasogastric tube was used for feeding. It could be removed after full oral feeding was secured after 7 days. The postoperative course was uneventful. Pre- and postoperative logopedic assessment of fiber optic endoscopic evaluation of swallowing (FEES) and functional oral intake score as well as voice assessment did not show any differences between the pre- and the postoperative examination.

Final pathology showed SCC with a horizontal extension of max.: 8 mm, depth of invasion of max 3 mm. The closest resection margins were left 4 mm, right 5 mm, anterior 10 mm, posterior 4 mm, deep 5 mm; and hyoid bone free of disease. No lymphovascular invasion was noted. The patient remained free of disease locally at last follow-up about 20 months after surgery.

In conclusion, we show a novel technique for improvement of TORS exposure for BOT resection and allowing sufficient deep “bony” margins to the hyoid bone.
